# Gas hydrate inhibition by perturbation of liquid water structure

**DOI:** 10.1038/srep11526

**Published:** 2015-06-17

**Authors:** Jeong-Hoon Sa, Gye-Hoon Kwak, Kunwoo Han, Docheon Ahn, Kun-Hong Lee

**Affiliations:** 1 Department of Chemical Engineering, Pohang University of Science & Technology, 77 Cheongam-Ro, Nam-Gu, Pohang, Gyeongbuk 790-784, Korea; 2 CO2 Project Team, Research Institute of Industrial Science & Technology, 67 Cheongam-Ro, Nam-Gu, Pohang, Gyeongbuk 790-600, Korea; 3 Beamline Division, Pohang Accelerator Laboratory, 80 Jigok-Ro 127Beon-Gil, Nam-Gu, Pohang, Gyeongbuk 790-834, Korea

## Abstract

Natural gas hydrates are icy crystalline materials that contain hydrocarbons, which are the primary energy source for this civilization. The abundance of naturally occurring gas hydrates leads to a growing interest in exploitation. Despite their potential as energy resources and in industrial applications, there is insufficient understanding of hydrate kinetics, which hinders the utilization of these invaluable resources. Perturbation of liquid water structure by solutes has been proposed to be a key process in hydrate inhibition, but this hypothesis remains unproven. Here, we report the direct observation of the perturbation of the liquid water structure induced by amino acids using polarized Raman spectroscopy, and its influence on gas hydrate nucleation and growth kinetics. Amino acids with hydrophilic and/or electrically charged side chains disrupted the water structure and thus provided effective hydrate inhibition. The strong correlation between the extent of perturbation by amino acids and their inhibition performance constitutes convincing evidence for the perturbation inhibition mechanism. The present findings bring the practical applications of gas hydrates significantly closer, and provide a new perspective on the freezing and melting phenomena of naturally occurring gas hydrates.

Gas hydrates are ice-like solids in which gases are enclosed in a hydrogen-bonded water crystal lattice^1^, and have attracted worldwide attention as potential clean energy resources because of their natural abundance^2^. The amount of hydrocarbons stored in naturally occurring gas hydrates approaches that of all other fossil fuel reserves in the world combined^3^. Gas hydrates form under low temperature and high pressure conditions, so are usually found under marine sediments and permafrost. The Arctic region contains substantial natural gas and oil reserves^4^, as well as mineral resources and fisheries^5^, so the increased availability of sea routes has resulted in increased interest in Arctic exploration^6^.

Gas hydrate formation and inhibition phenomena are also important to gas and oil industry. Natural gas transportation lines often provide thermodynamic conditions that are favorable for gas hydrate formation^7^. In addition, a massive increase in the amount of CO_2_ emission from fuel combustions leads to a growing interest in CO_2_ transportation through pipelines. Unfortunately, this process sometimes causes pipeline blockages, which leads to unavoidable delays in operation, the need for repairs, and severe financial losses. A variety of hydrate prevention methods^1^ such as thermal heating, depressurization, and mechanical elimination have been suggested; one simple method is the injection of hydrate inhibitors^1^. The injection of thermodynamic hydrate inhibitors such as alcohols and glycols is regarded as a robust method, since these compounds permanently inhibit hydrate formation by directly changing the temperature and pressure conditions of formation. However, this method requires the injection of huge amounts of these compounds, so the use of kinetic hydrate inhibitors (KHIs) has been suggested as a promising alternative because they can delay hydrate nucleation and growth at much lower concentrations^8^. One significant issue for the utilization of KHIs is the prediction of when gas hydrates will nucleate, and then how quickly they will grow. Despite strenuous efforts, gas hydrate inhibition phenomena are still poorly understood because of their inherent complexity.

It was originally thought that KHIs adsorb directly onto the hydrate surface. The morphologies of hydrate crystals were found experimentally to be changed by the presence of KHIs such as polymers^9^,^10^, antifreeze proteins^11^, and quaternary ammonium zwitterions^12^. In addition, Monte Carlo^13^ and molecular dynamics^14^,^15^ simulations have been performed to investigate the formation of hydrogen bonds between oxygen species in KHIs and hydrogen on hydrate surfaces. However, other studies have disputed this mechanism^16^,^17^,^18^,^19^ because it was found that some KHIs do not make direct contact with hydrate surfaces. Several more recent studies have proposed that perturbation of the structure of liquid water by KHIs is another mechanism that can be used to interpret gas hydrate inhibition phenomena^12^,^15^. In general, such perturbation is a universal phenomenon in which a solute dissolved in water induces changes in the structure of water^20^,^21^,^22^,^23^. Simulation results have showed that the hydrophilic moieties of KHIs disrupt the structure of water and render it incompatible with the hydrate structure, and that this disruption is responsible for hydrate inhibition^12^. However, there have been almost no experimental assessments of this hypothesis. Our recent study proposed that the KHI abilities of some hydrophobic amino acids with respect to CO_2_ hydrate inhibition are attributed to the perturbation of the structure of the surrounding water^24^, but no experimental evidence for this proposal has yet been reported.

In this study, we directly observed the perturbation of the structure of liquid water by amino acids, and investigated the influence of this perturbation on CO_2_ hydrate nucleation and growth kinetics. The findings of this study provide strong experimental evidence in support of the hypothesis that perturbation plays a critical role in the inhibition of gas hydrate formation.

## Results and discussion

### Model amino acid system

The five amino acids alanine, aspartic acid, asparagine, phenylalanine, and histidine were tested as solutes (Table 1) as they have distinct side chains in their molecular structures. If we think of alanine as the basis of this system, aspartic acid, asparagine, phenylalanine, and histidine have additional carboxylic acid (−COOH), amide (−CONH_2_), phenyl (−C_6_H_5_), and imidazole (−C_3_H_3_N_2_) groups, respectively. The hydrophobicity, acidity, and solubility of an amino acid in water strongly depend on its side chain properties. Thus, we can vary the physicochemical properties of the test compound by simply selecting a suitable molecule.

An important consideration in the investigation of the interactions of an amino acid side chain with water molecules is the charge distribution within the amino acid molecule. Although the carboxylic acid and amine groups that are commonly attached to the central carbon atoms of amino acids can form carboxylate and ammonium ions respectively, the electric charge distributions of such side chains are entirely different from those of the respective ions (see Supplementary Fig. S1). In particular, the hydrophilic nature of the side chains and their ability to form ionized or resonance structures induces polarization of the amino acids (Supplementary information). The molecular polarization of an amino acid is expected to increase the strength of its interactions with water molecules, and hence the extent of the perturbation of the structure of liquid water.

### Perturbation of liquid water structure

**Figure 1:**
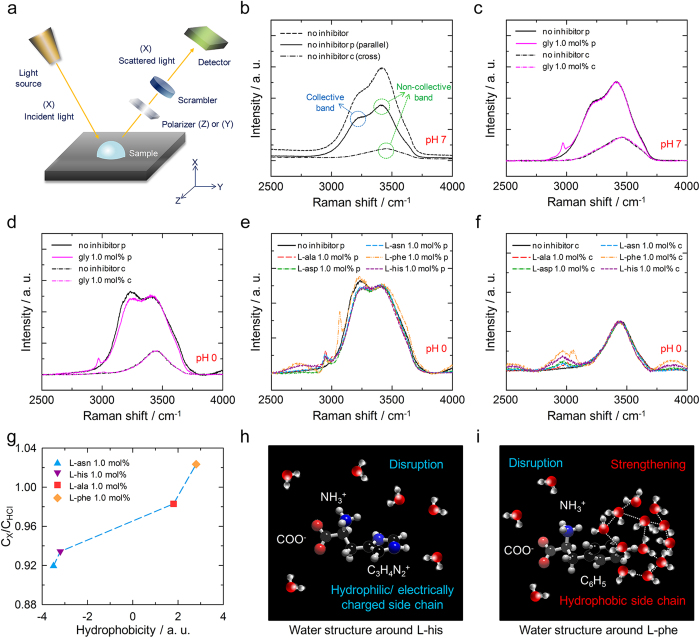
Perturbation of liquid water structure by amino acids. (**a**) Experimental set-up for performing polarized Raman spectroscopy. (**b**) Raman and polarized Raman spectra of pure liquid water. The OH stretching band near 3250 cm^−1^ was due to collective motion of hydrogen-bonded water. Polarized Raman spectra of an aqueous glycine solution (**c**) under neutral conditions and (**d**) under acidic conditions. Polarized Raman spectra of the aqueous amino acid solutions under acidic conditions (**e**) for the parallel position and (**f**) for the cross position. The amino acids affected the collective motion of water. (**g**) Correlation between the extents of perturbation due to the amino acids and their hydrophobicities. Decreases and increases in *C*_*X*_*/C*_*HCl*_ indicate disruptions and strengthenings respectively of the water structure. Hypothetical representations of the liquid water structure (**h**) around L-histidine and (**i**) around L-phenylalanine. The hydrophilic and electrically charged side chain of L-histidine disrupted the water structure, whereas the hydrophobic side chain of L-phenylalanine strengthened it.

To examine the perturbations of liquid water structure by amino acids, polarized Raman spectra of aqueous solutions of the amino acids were obtained. In these experiments, scattered light from the aqueous amino acid solution passed through a polarizer plate and a scrambler (Fig. 1a); this experimental set-up is very similar to that used in previous studies^25^,^26^,^27^. The polarization geometries were X(ZZ)X and X(ZY)X for the parallel and cross positions respectively. In the Raman spectrum of pure liquid water (no inhibitor), two broad bands centered near 3250 and 3400 cm^−1^ were observed (Fig. 1b), which were attributed to symmetric and asymmetric OH stretching respectively^28^,^29^. When a polarizer was applied to extract the polarized Raman spectra for the parallel and cross positions, only the lower frequency band was highly polarized. This lower frequency band has previously been assigned to the collective in-phase stretching motion of strongly hydrogen-bonded water^30^,^31^, and is thus due to the intrinsic dynamics of liquid water molecules^32^,^33^. The ratio of the intensity of this band to that of the non-collective band can be used to quantify the fraction of such water^34^. As this ratio increases, the water molecules become more strongly connected to each other, whereas a decrease in this ratio indicates the disruption of the hydrogen bond network^25^,^26^.

On the addition of 1.0 mol% glycine to water, the two broad OH stretching bands in both the parallel and cross spectra were only slightly affected (Fig. 1c), consistent with a previous report^27^, and an additional band due to the CH_2_ symmetric stretching of glycine near 2970 cm^−1^ was present^35^. This result has been interpreted as follows: the interaction of the amino acid side chains with water is shielded by large overlapping hydration shells around the ammonium and carboxylate ions, and the accessibility of water to the side chains is considerably reduced by the self-association of amino acids via electrostatic interactions at neutral pH^27^. To investigate the influence of the amino acid side chains on the structure of water, an aqueous HCl solution was used to induce the deionization of the carboxylate ions. An acidic environment is also close to the actual conditions for CO_2_ hydrate formation due to the presence of carbonic acids in water^36^. An increase in CO_2_ pressure accelerates the formation of carbonic acids, thus significantly decreasing the pH. According to the previous report, the pH values of the CO_2_ hydrate system can be decreased to around 3.18 with 35 bar of CO_2_ at 283.15 K^36^. Under acidic conditions, the addition of 1.0 mol% glycine reduced the intensity of the collective band, indicating the disruption of the water structure (Fig. 1d). Since glycine has no side chain, it seems likely that the carboxylate and ammonium ions produced this disruption of the water hydrogen bond network owing to their hydrophilic nature and electric charges.

These experiments were repeated with the solutions containing various amino acids. The effects of the presence of the amino acids on the parallel spectra varied with the properties of their side chains (Fig. 1e); the OH stretching bands in the cross spectra were not affected (Fig. 1f). L-alanine, L-aspartic acid, L-asparagine, and L-histidine were found to definitely reduce the intensity of the collective band, indicating the disruption of the water structure. In contrast, L-phenylalanine, the most hydrophobic of the tested amino acids, was found to increase the band intensity by strengthening the hydrogen bond network. To quantify the extent of each perturbation, the *C* value of each parallel spectrum was calculated from the ratio of the intensities of the collective band and the non-collective band. A *C* value larger than 1 indicates the strengthening of the water structure, whereas a value less than 1 indicates disruption. The relative *C* values of the aqueous HCl solutions containing amino acids with respect to that of HCl solution were plotted against the hydrophobicities of the amino acids (Fig. 1g). The extent of perturbation was strongly correlated with the hydrophobicity, consistent with a previous report^27^. This trend clearly indicates that hydrophilic and/or electrically charged side chains disrupt the water structure, as depicted (Fig. 1h). While L-phenylalanine also contains carboxylate and ammonium ions, the strengthening of the structure of water around the hydrophobic side chains would be greater than the disruption around the ionic moieties (Fig. 1i).

These experimental results demonstrate that the structure of liquid water is perturbed by the presence of the amino acids, and that the extent of perturbation varies with the side chain properties. Therefore, the amino acids tested were found to be suitable for the investigation of the influence of perturbation on hydrate inhibition.

### Heterogeneous nucleation kinetics

**Figure 2:**
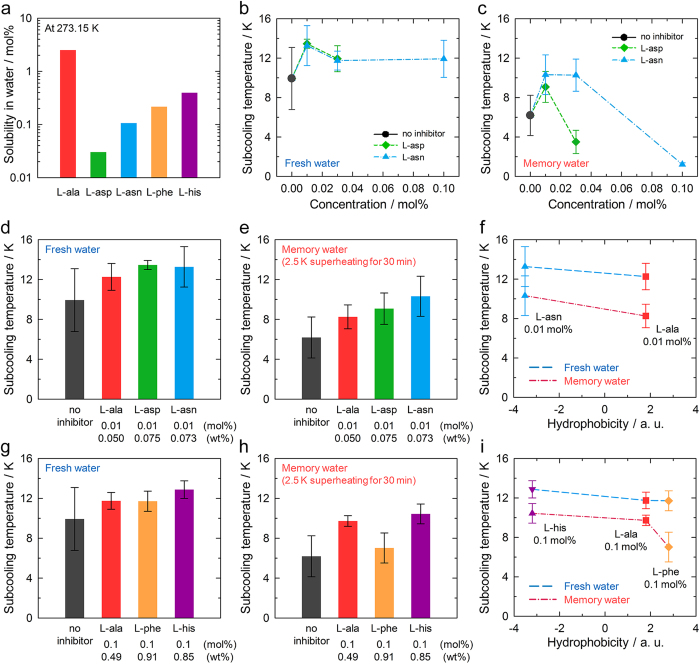
Heterogeneous nucleation kinetics of the CO_2_ hydrates. (**a**) The solubilities of the amino acids in water at 273.15 K^42^,^43^. CO_2_ hydrate nucleation kinetics (**b**) in fresh water and (**c**) in memory water. Memory water was obtained from the dissociation of CO_2_ hydrate at a temperature 2.5 K above the phase equilibrium temperature for 30 min. The injection of KHIs at concentrations closed their solubilities led to failures in hydrate inhibition. CO_2_ hydrate nucleation kinetics in the presence of 0.01 mol% amino acids (**d**) in fresh water and (**e**) in memory water. (**f**) The correlation of subcooling temperatures at the onset of CO_2_ hydrate nucleation with the hydrophobicities. CO_2_ hydrate nucleation kinetics in the presence of 0.1 mol% amino acids (**g**) in fresh water and (**h**) in memory water. (**i**) The correlation of subcooling temperatures with the amino acid hydrophobicities. The values for the average and standard deviation are shown. L-phenylalanine had negligible influence on nucleation kinetics, especially in memory water, whereas all the other tested amino acids were found to be effective in inhibiting CO_2_ hydrate nucleation. The data for the system with no inhibitor or 0.1 mol% L-alanine were obtained from our previous report^24^.

To examine the effects of the amino acids on hydrate nucleation, the subcooling temperature, which is the difference between the onset temperature and the phase equilibrium temperature at the onset pressure, was obtained for each amino acid (Supplementary information). Hydrate nucleation is accompanied by a sharp increase in temperature during cooling, as described in our previous report^24^. When investigating heterogeneous nucleation kinetics, an important consideration is the solubility of the KHI in water under hydrate forming conditions. The solubility values of the tested amino acids in water at 273.15 K were plotted (Fig. 2a); this temperature was chosen as it is the temperature with the lowest solubility values. On increasing the concentrations of L-aspartic acid and L-asparagine, the nucleation kinetics did not change significantly (Fig. 2b). The measurements were repeated by using memory water with a previous hydrate history^1^, since this method gives more reliable results^37^. Memory water was prepared by dissociating the hydrates, and thus having a thermal hysteresis for hydrate formation. Therefore, hydrate nucleation occurs much easier in the memory water. In the memory water system, however, as the concentrations of L-aspartic acid and L-asparagine exceeded their solubilities, the subcooling temperature values suddenly dropped (Fig. 2c), indicating the nucleation kinetics was accelerated. This failure of hydrate inhibition may be attributed to the presence of undissolved amino acids that precipitated during hydrate dissociation, which could provide additional sites for heterogeneous hydrate nucleation. The inhibition performances of the amino acids were therefore measured at concentrations much lower than their solubility values.

In the measurements of the heterogeneous nucleation kinetics, the subcooling temperatures of the solutions containing amino acids were about 2–4 K higher than those of the solution without inhibitor (Fig. 2d,e), indicating that L-alanine, L-aspartic acid, and L-asparagine delay hydrate nucleation. Interestingly, L-aspartic acid and L-asparagine were found to be more effective in hydrate inhibition than L-alanine, and their inhibition performances were correlated with their hydrophobicities (Fig. 2f). This correlation is attributed to the disruption of the water structure by their hydrophilic side chains, as indicated by the Raman spectra. As expected, L-histidine was also found to be more effective in hydrate inhibition than L-alanine, owing to its hydrophilic side chain (Fig. 2g,h). However, L-phenylalanine had almost no influence on the kinetics of hydrate nucleation, especially in memory water. The structure of water around the hydrophobic L-phenylalanine side chain was strengthened, even though the structure was disrupted around the carboxylate and ammonium ions. The competition between the disruption and strengthening of the water structure can affect the nucleation kinetics. The inhibition performances of L-alanine, L-phenylalanine, and L-histidine were strongly correlated with their hydrophobicities (Fig. 2i). It is obvious that the nucleation inhibition performances of the amino acids increased with decreasing hydrophobicity, with a similar trend to that found in our previous study^24^. Surprisingly, this trend is also very similar to the trend in the extent of perturbation obtained from the polarized Raman spectra (Fig. 1g). The strong correlation of the nucleation inhibition performances of the amino acids with the extents of their perturbations of water structure suggests that water structure is an important factor in hydrate nucleation. Amino acids with hydrophilic and/or electrically charged side chains are likely to be effective in delaying hydrate nucleation because of their disruptions of water structure, whereas those with hydrophobic side chains have negligible effects on kinetics due to the competition between the strengthening of the water structure around side chains and the disruption of the water structure around ionic moieties.

### Growth kinetics

**Figure 3:**
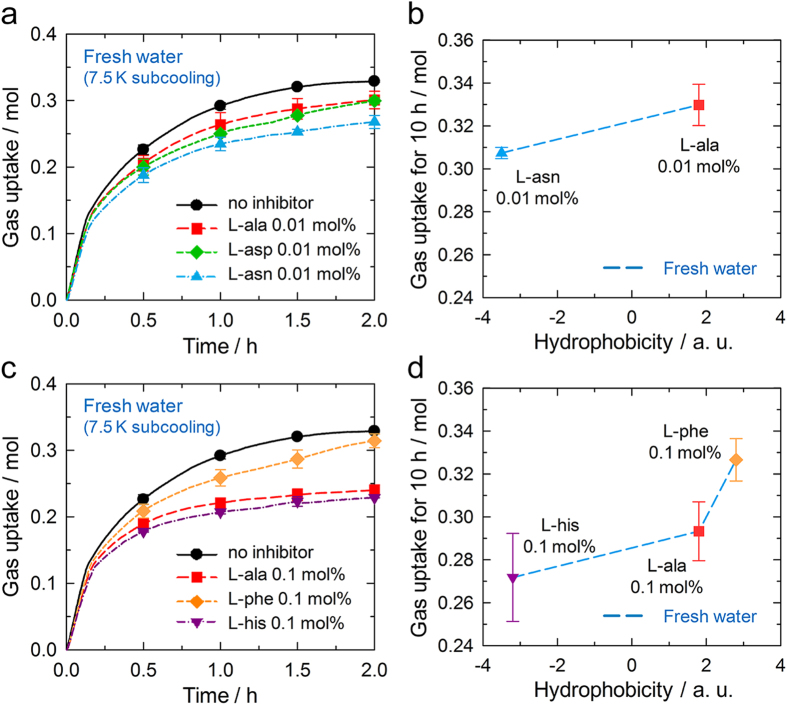
Growth kinetics of the CO_2_ hydrates. (**a**) CO_2_ hydrate growth kinetics in the presence of 0.01 mol% amino acids in fresh water. The gas uptake measurements were initiated at the onset of CO_2_ hydrate nucleation. (**b**) The correlation between the amount of gas uptake over 10 h and amino acid hydrophobicity. (**c**) CO_2_ hydrate growth kinetics in the presence of 0.1 mol% amino acids in fresh water. (**d**) The correlation of gas uptake values with the amino acid hydrophobicities. The lines are the average values of the gas uptake, which were obtained every 10 s. The symbols and their error bars indicate the average and standard deviation, respectively. All the tested amino acids were found to inhibit CO_2_ hydrate growth. The data for the solutions with no inhibitor or 0.1 mol% L-alanine were obtained from our previous report^24^.

Further investigations of the kinetics of hydrate growth were performed. In the solution without inhibitor, rapid growth was initiated immediately after the onset of hydrate nucleation, and the growth rate gradually decreased over time (Fig. 3a). On the addition of 0.01 mol% amino acids, however, an inflection point was observed after the rapid growth over the first 7 min, and then the growth rate fell below that of the solution without inhibitor. L-aspartic acid and L-asparagine exhibited better growth inhibition performances than L-alanine, which can be attributed to their hydrophilic side chains. Their growth inhibition performances were also correlated with their hydrophobicities (Fig. 3b). The addition of 0.1 mol% amino acids produced similar results (Fig. 3c). These experimental results show that these amino acids delayed hydrate growth as well as hydrate nucleation. Although L-phenylalanine also reduced the rate of hydrate growth, it was less effective than L-alanine owing to its hydrophobic side chain. The ability to retard hydrate growth was strongly correlated with the hydrophobicity (Fig. 3d), consistent with the results of our previous studies^24^,^38^. All of the tested amino acids were found to delay hydrate growth.

An intriguing point is that the trend in the growth inhibition performances of the amino acids was very similar to that found for the heterogeneous nucleation kinetics. It is especially noteworthy in that there have been several previous reports of discrepancies between the nucleation and growth inhibition performances of KHIs^39^,^40^. These discrepancies have been attributed to the fact that hydrate growth occurs on hydrate surfaces, whereas heterogeneous hydrate nucleation mostly initiates from solid nucleating impurities^1^. Therefore, it is likely that the effects of amino acids are independent of surface properties. These findings imply that perturbation is a better explanation for hydrate inhibition by amino acids than adsorption onto the hydrate surface, because perturbation arises in the presence of KHIs due to the interruption of hydrogen bonds between water molecules and not the presence of any surface. In addition, the growth inhibition performances of the amino acids were found to be closely related to the extent of perturbation. This correlation strongly supports the perturbation inhibition mechanism.

## Conclusion

In summary, we have directly observed perturbation of the structure of liquid water by amino acids, and investigated the influence of this perturbation on the kinetics of gas hydrate nucleation and growth. Amino acids with hydrophilic and/or electrically charged side chains were found to disrupt the structure of liquid water, whereas those with hydrophobic side chains strengthened this structure. The KHI efficacies of the amino acids were strongly correlated with their hydrophobicities, and thus with the extents of perturbation. These findings indicate that perturbation phenomena play a vital role in hydrate inhibition, and also imply that any molecule dissolved in water will affect the structure of water and hydrate kinetics because of its intrinsic hydrophobic or hydrophilic nature. The fundamental insights into gas hydrate kinetics and the mechanism of hydrate inhibition gained in this study are beneficial for understanding the freezing and melting phenomena of gas hydrates. In addition, the present findings will assist a variety of industrial applications including gas storage, separation, and transportation.

## Methods

### Polarized Raman spectroscopy

The experimental set-up for the polarized Raman spectroscopy measurements is presented (Fig. 1a). LabRam Aramis (Horiba Jobin Yvon) Raman spectroscopy was performed under ambient conditions. The incident light was provided by a 514 nm Ar-ion laser with 2 mW power. A scrambler was used to remove instrumental errors. A droplet of each aqueous amino acid solution (0.1 mL) was loaded onto a polymer holder covered with aluminum foil, and the scan was performed from 2500 cm^−1^ to 4000 cm^−1^ for 60 s with a band resolution of 2 cm^−1^. The baselines were adjusted, and then all spectra were normalized.

In previous studies^25^,^26^,^27^, the extent of perturbation has been calculated with an area-based method. The intensity of the collective band is extracted from the intensity of the polarized Raman spectra for the parallel and cross positions. Then, the *C* value is calculated through integration of the intensity of the spectra, and then normalized. However, this method can produce erroneous results. An adjustment is involved in the extraction of the collective band because the difference spectrum obtained from the polarized Raman spectra for the parallel and cross positions is very sensitive to the degree of depolarization. In addition, the resulting spectrum of the collective band is assumed to be symmetric, and the entire spectrum is defined by the results for the low-frequency region.

In this study, we quantified the extent of perturbation by using an intensity-based method. The key idea of this method is very similar to that of the previous method in that the *C* value is obtained from the difference between the intensities of the collective and non-collective bands. This method simply uses the maximum band intensity, and thus does not distort the raw experimental data. The *C* value is defined by the following equation (1):


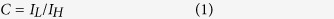


where *I*_*L*_ is the maximum intensity of the lower frequency band (~3250 cm^−1^) in the parallel component, and *I*_*H*_ is that of the higher frequency band (~3400 cm^−1^). The lower frequency and higher frequency bands correspond to the collective and non-collective bands respectively. Then, the relative *C* value of an aqueous amino acid solution is defined as follows:


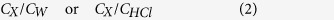


where *C*_*X*_ is the *C* value of the aqueous amino acid solution, *C*_*W*_ is that of pure water, and *C*_*HCl*_ is that of the aqueous HCl solution.

## Additional Information

**How to cite this article**: Sa, J.-H. *et al.* Gas hydrate inhibition by perturbation of liquid water structure. *Sci. Rep.* **5**, 11526; doi: 10.1038/srep11526 (2015).

## Supplementary Material

Supplementary Information

## Figures and Tables

**Table 1:**
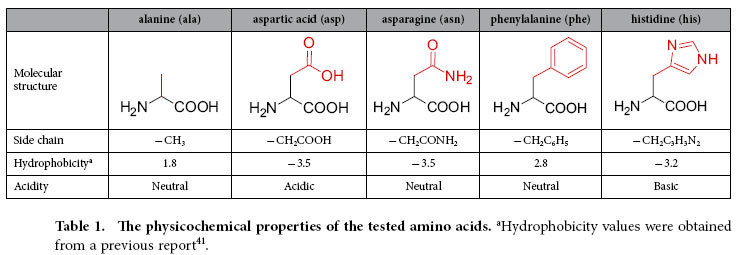
The physicochemical properties of the tested amino acids.
