# Supplementary material for: Gas hydrate inhibition by perturbation of liquid water structure
Source: Sci Rep. 2015 Jun 17;5:11526. doi: 10.1038/srep11526 (PMC4469970; doi:10.1038/srep11526)
Supplement: Supplementary Information [file srep11526-s1.doc]

**Supplementary information for:**

**Gas hydrate inhibition by perturbation of liquid water structure**

Jeong-Hoon Sa,^1^ Gye-Hoon Kwak,^1^ Kunwoo Han,^2^ Docheon Ahn,^3^ and Kun-Hong Lee^1*^

^1^*Department of Chemical Engineering, Pohang University of Science & Technology,*

*77 Cheongam-Ro, Nam-Gu, Pohang, Gyeongbuk 790-784, Korea*

^2^*CO_2_ Project Team, Research Institute of Industrial Science & Technology,*

*67 Cheongam-Ro, Nam-Gu, Pohang, Gyeongbuk 790-600, Korea*

^3^*Beamline Division, Pohang Accelerator Laboratory,*

*80 Jigok-Ro 127Beon-Gil, Nam-Gu, Pohang, Gyeongbuk 790-834, Korea*

*Corresponding author. Tel: 82-54-279-2271; Fax: 82-54-279-8298; e-mail: [ce20047@postech.ac.kr](mailto:ce20047@postech.ac.kr)

**1. Experimental**

**Materials**

Distilled water was prepared in our laboratory. 1.0 M HCl solution was purchased from Sigma-Aldrich, USA. Amino acids (glycine, L-alanine, L-aspartic acid, L-asparagine, L-phenylalanine, and L-histidine) were also supplied by Sigma-Aldrich, USA. All amino acids were of reagent grade (≥99%). All chemicals were used without further purification. Ultra-high purity CO_2_ (99.999%) was obtained from Deokyang Energen Corporation, Korea.

**Heterogeneous nucleation kinetics**

The experimental set-up for kinetics measurement was as described in our previous report.^1^ Two high pressure cells made of SUS 315 with a volume of 250 cm^3^ were used. The cell contents were mixed by using an impeller coupled with a magnetic drive. The cell temperature was obtained with a K-type thermocouple with a maximum error of 0.1 K. The cell pressure was measured by using a WIKA A-10 pressure transducer with a maximum error of 0.5%. Each amino acid was dissolved in 60 g water, and then charged in a cell. The assembled cell was immersed in an ethanol bath, and charged with 36 bar of CO_2_ at 284.05 K. The cell contents were mixed with agitation at 450 rpm.

The constant cooling method was used to measure the nucleation kinetics in fresh water, as described in our previous report.^1^ The cell was cooled at the maximum cooling rate (0.25 K/min). Upon cooling, hydrate nucleation was detected by monitoring for a dramatic increase in temperature. The subcooling temperature is defined as the difference between the onset temperature and the phase equilibrium temperature at the onset pressure.

Constant cooling with the superheated hydrate method was used to measure the nucleation kinetics in memory water, as described in our previous report.^1^ When the cell pressure was lowered to approximately 5 bar below the onset pressure, agitation was stopped and the cell temperature was rapidly increased to 283.95 K, which is 2.5 K higher than the phase equilibrium temperature, for 15 min. When the pressure reached 35 bar, agitation was resumed for 30 min to induce complete hydrate dissociation. After these procedures, the characterization of the hydrate nucleation kinetics was repeated following the constant cooling method.

**Growth kinetics**

The isothermal method was used to characterize the nucleation kinetics in fresh water, as described in our previous report.^1^ The system was cooled to 273.45 K without agitation. When the temperature reached 273.45 K, agitation at 450 rpm was employed to induce hydrate nucleation. After hydrate nucleation, the rate of hydrate growth was measured at a constant temperature. The gas uptake rate was calculated with the following equation (1).^3^

$\Delta n_{h}=n_{h,t}-n_{h,0}=\left(\frac{PV}{ZRT}\right)_{0}-\left(\frac{PV}{ZRT}\right)_{t}$ (1)

where *n_h_* is the amount of gas molecules consumed during hydrate formation at times *t* and 0, *Z* is the compressibility factor calculated with Pitzer’s correlation,^4^ and *V* is the cell volume of the vapor phase.

**Synchrotron powder X-ray diffraction (PXRD)**

PXRD experiments were carried out at the Pohang Accelerator Laboratory. The details of these experiments are presented in our previous report.^1,2^ The incident X-rays were monochromatized to a wavelength of 1.54740 Å by using a silicon (111) crystal. A 1.0 g powder sample was loaded onto a flat plate holder and the scan was performed at 80.0 K with a fixed time step of 1 s and a step size in 2θ of 0.005° from 10° to 130.5° with a 0.5° overlap. The lattice parameter values of the hydrate samples were calculated from the powder X-ray diffraction patterns by performing two-phase (structure I hydrate + hexagonal ice) full pattern (10° to 110.5°) matching with the FullProf program.^5^

**2. Ionization and electron delocalization of the amino acid side chains**

Amino acid side chains have distinctive features especially in aqueous environments. The negative charge on the carboxylate ion in the aspartic acid side chain is delocalized over two oxygen atoms, thereby stabilizing the ions and enhancing its acidity (Fig. S1a). Curiously, although asparagine is electrically neutral, its amide side chains also have resonance forms, in which the lone pair of electrons on the nitrogen atom is delocalized into the carbonyl group (Fig. S1b). This feature means that partial electric charges are present in the asparagine molecule. In contrast, the delocalization of π electrons within the phenyl ring induces barely any polarization, since all these carbon-carbon bonds are almost equivalent (Fig. S1c). Thus, phenylalanine is classified as hydrophobic amino acid due to the hydrophobic character of its benzyl side chain. Further, the mode of electron delocalization along the side chain of histidine varies with the pH, since the two nitrogen atoms in the aromatic imidazole ring are not equivalent. Under neutral conditions, only a lone pair of electrons on the pyrrole-like singly bonded nitrogen atom is delocalized into the ring, whereas pyridine-like doubly bonded nitrogen atom is basic. However, the basic nitrogen is protonated under acidic conditions, so the positive charge is delocalized over the two nitrogen atoms (Fig. S1d).


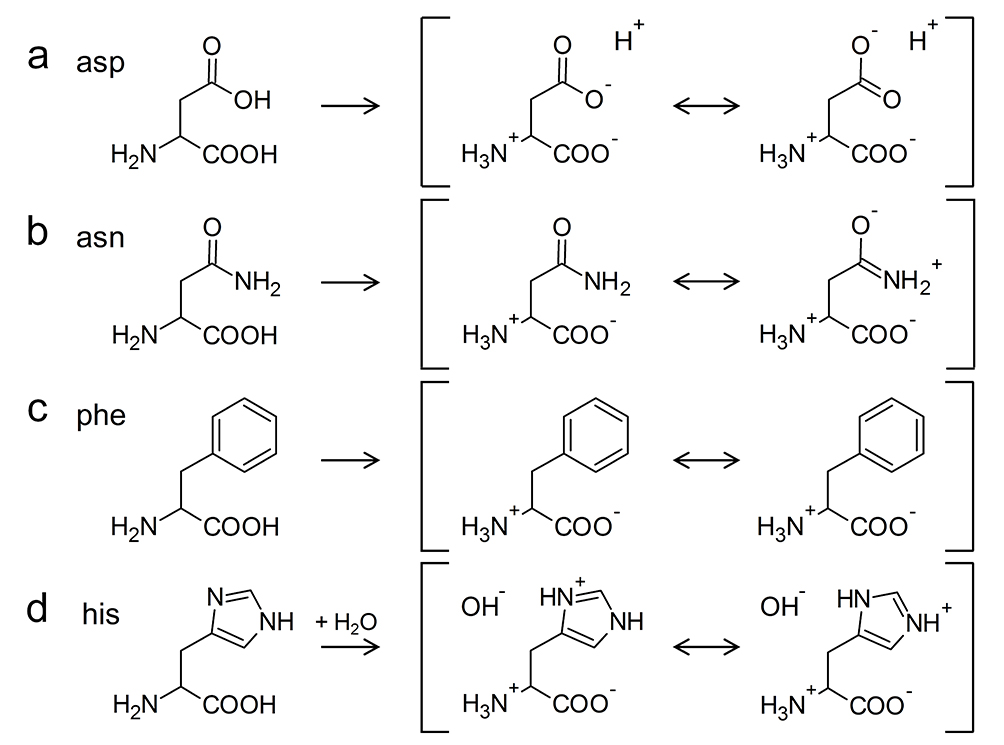


**Figure S1. Ionization and electron delocalization of (a) carboxylic acid, (b) amide, (c) phenyl, and (d) imidazole groups in an aqueous environment.**

**3. Growth kinetics**

The growth kinetics for 10 h of hydrate formation are shown in Fig. S2. Although there is negligible increase in the gas uptake for most of the solutions in the period of 2 to 10 h, there are marked increases in others.


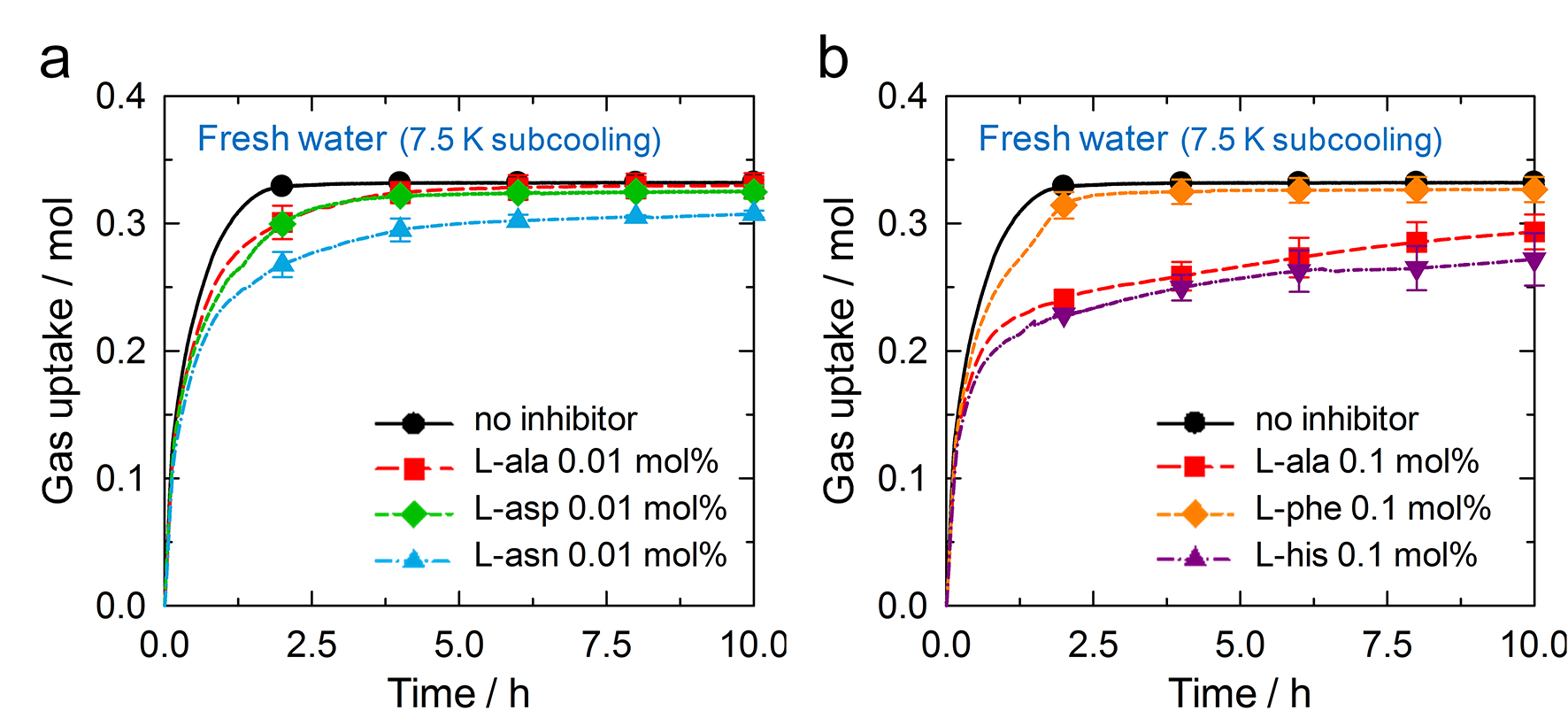


**Figure S2. Growth kinetics of the CO_2_ hydrates in the presence of (a) 0.01 mol% amino acids and (b) 0.1 mol% amino acids.** Measurement of hydrate growth was initiated just after the onset of hydrate nucleation. The average values of the gas uptake were calculated from several repeated measurements obtained every 10 s. The symbols and their error bars indicate the average and standard deviations respectively of the gas uptake at each time point. Data for the solution without inhibitor and containing 0.1 mol% L-alanine were obtained from our previous report.^1^

**4. PXRD**

All samples were found to have cubic structure I hydrate with a *Pm3n* and hexagonal ice phase with a *P6_3_/mmc* space group by using the FullProf program.^5^ When the amino acids are added, the number of diffraction peaks for the ice phase slightly increases. However, these negligible differences indicate that amino acids are not effective in reducing the amount of hydrates after a long period of hydrate formation.


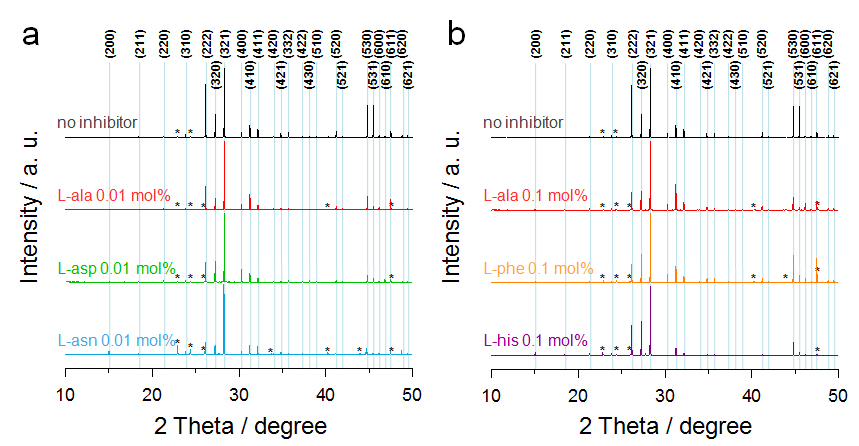


**Figure S3. Synchrotron PXRD patterns of the CO_2_ hydrates in the presence of (a) 0.01 mol% and (b) 0.1 mol% amino acids.** The diffraction peaks for the structure I hydrates are denoted by their Miller indices, and the asterisks indicate the peak positions for the ice phase.

**5. Lattice parameter calculation**

To calculate the lattice dimensions, profile matching was performed for the PXRD patterns of all samples by using the FullProf program.^5^ The results for CO_2_ hydrate with the addition of 0.1 mol% L-alanine are shown (Fig. S4).


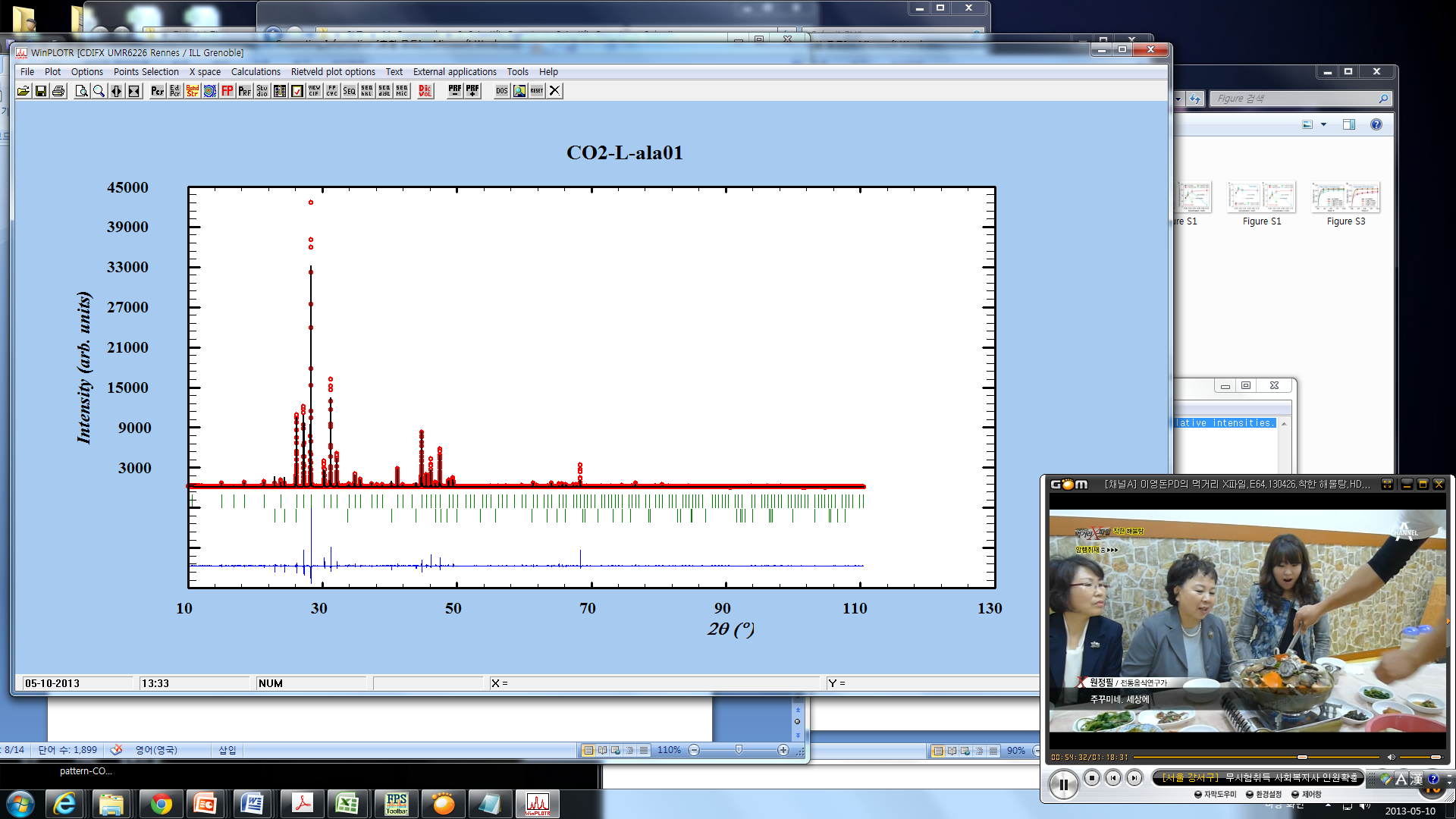


**Figure S4. Profile matching result for the diffraction pattern of the CO_2_ hydrate in the presence of 0.1 mol% L-alanine at 80 K.** The red circular spots are the observed intensities, and the black solid line was calculated from the profile matching. The upper and lower green tick marks show the calculated peak positions for the structure I hydrate and the ice phase respectively. The blue line is the difference between the observed and calculated intensities. (*R*_p_ = 13.7%, *R*_wp_ = 18.7%, *R*_exp_ = 9.45%, *S* = 1.98, *χ*^2^ = 3.92)

The lattice parameter and volume values for all samples were calculated from the diffraction patterns by using the FullProf program.^5^ It was confirmed that the addition of the amino acids does not alter the crystal structure of the CO_2_ hydrates significantly.

**Table S1.** Calculated lattice parameters and volumes for CO_2_ hydrates in the presence of amino acids.

| System | Lattice parameter (Å) | Lattice volume (Å^3^) |
| --- | --- | --- |
| no inhibitor | 11.844508 ± 0.000052 | 1661.6941 ± 0.0127 |
| L-ala 0.01 mol% | 11.843655 ± 0.000059 | 1661.3351 ± 0.0143 |
| L-asp 0.01 mol% | 11.858191 ± 0.000186 | 1667.4596 ± 0.0454 |
| L-asn 0.01 mol% | 11.844112 ± 0.000086 | 1661.5274 ± 0.0209 |
| L-ala 0.1 mol% | 11.845165 ± 0.000065 | 1661.9706 ± 0.0158 |
| L-phe 0.1 mol% | 11.842922 ± 0.000055 | 1661.0267 ± 0.0133 |
| L-his 0.1 mol% | 11.841277 ± 0.000169 | 1660.3346 ± 0.0410 |

**References**

1. Sa, J.-H. *et al*. Hydrophobic amino acids as a new class of kinetic inhibitors for gas hydrate formation. *Sci. Rep.* **3**, 2428 (2013).
2. Sa, J.-H., *et al*. Abnormal incorporation of amino acids into the gas hydrate crystal lattice. *Phys. Chem. Chem. Phys.* **16**, 26730-26734 (2014).
3. Linga, P., Kumar, R. & Englezos, P. Gas hydrate formation from hydrogen/carbon dioxide and nitrogen/carbon dioxide gas mixtures. *Chem. Eng. Sci.* **62**, 4268–4276 (2007).
4. Smith, J. M., Van Ness, H. C. & Abbott, M. M. *Introduction to Chemical Engineering Thermodynamics* (McGraw-Hill, New York, 2001).
5. Rodrigues-Carvajal, J. Recent advances in magnetic structure determination by neutron powder diffraction. *Physica B* **192**, 55-69 (1993).
